# Long-term trends in population-based hospitalisation rates for myocardial infarction in England: a national database study of 3.5 million admissions, 1968–2016

**DOI:** 10.1136/jech-2021-216689

**Published:** 2021-07-12

**Authors:** F Lucy Wright, Nick Townsend, Melanie Greenland, Michael J Goldacre, Kate Smolina, Ben Lacey, Lee Nedkoff

**Affiliations:** 1 Unit of Health-Care Epidemiology, Nuffield Department of Population Health and Big Data Institute, University of Oxford, Oxford, UK; 2 Department for Health, University of Bath, Bath, UK; 3 School of Population and Global Health, The University of Western Australia, Perth, Western Australia, Australia; 4 School of Population and Public Health, The University of British Columbia, Vancouver, British Columbia, Canada; 5 Clinical Trial Service Unit and Epidemiological Studies Unit, Nuffield Department of Population Health and Big Data Institute, University of Oxford, Oxford, UK

**Keywords:** epidemiology, ischaemic heart disease, record linkage

## Abstract

**Aim:**

To analyse the timing and scale of temporal changes in rates of hospitalised myocardial infarction (MI) in England by age and sex from 1968 to 2016.

**Methods:**

MI admissions for adults aged 15–84 years were identified from electronic hospital data. We calculated age-standardised and age-specific rates, and examined trends using joinpoint.

**Results:**

From 1968 to 2016, there were 3.5 million admissions for MI in England (68% men). Rates increased in the early years of the study in both men and women, peaked in the mid-1980s (355 per 100 000 population in men; 127 in women) and declined by 38.8% in men and 37.4% in women from 1990 to 2011. From 2012, however, modest increases were observed in both sexes. Long-term trends in rates over the study period varied by age and sex, with those aged 70 years and older having the greatest and most sustained increases in the early years (1968–1985). During subsequent years, rates decreased in most age groups until 2010–2011. The exception was younger women (35–49 years) and men (15–34 years) who experienced significant increases from the mid-1990s to 2007 (range +2.1%/year to 4.7%/year). From 2012 onwards, rates increased in all age groups except the oldest, with the most marked increases in men aged 15–34 years (7.2%/year) and women aged 40–49 (6.9%–7.3%/year).

**Conclusion:**

Despite substantial declines in hospital admission rates for MI in England since 1990, the burden of annual admissions remains high. Continued surveillance of trends and coronary disease preventive strategies are warranted.

**What is already known on this subject**

- Population-based mortality rates from coronary heart disease and myocardial infarction have been declining in England and other countries since the 1980s.
- Age-standardised hospital admission rates for myocardial infarction have been declining since the 1980s in many developed countries, but there are limited data on very long-term trends for England.
- Little is known about detailed long-term age-specific and sex-specific hospital admission rates for myocardial infarction in England and other countries.

**What this study adds**

- In England between 1968 and 2016, age-standardised hospitalisation rates for myocardial infarction increased during the 1970s, peaked in the mid-1980s and declined by a third until 2011, after which there was a modest increase.
- In the earlier years, trends varied by sex and age, and during the subsequent years, rates declined overall in most age groups until 2010–2011.
- After 2011, rates increased in most age groups with the most marked increase in young women (35–49 years) and young men (15–34 years).

## Introduction

Myocardial infarction (MI) is an acute, severe manifestation of coronary heart disease (CHD). Trends in MI reflect changes in coronary risk factors in the population. Accordingly, studying temporal trends in MI is a valuable way to monitor the overall impact of success or failure in controlling CHD risk and to anticipate likely future trends in MI. CHD mortality rates increased in the early to mid-20th century, and have declined since the 1980s, in most developed countries.^1 2^ In England, too, these latter downward trends have been seen for deaths from CHD,^2 3^ and specifically from MI.^4 5^ Despite this, CHD remains a major cause of morbidity,^6^ and it costs England’s National Health Service (NHS) over £950 million per year.^2^

Hospital admission rates for MI are an important measure of CHD burden in the population, but have generally been reported in studies covering relatively short time periods. Examination of long-term trends enables better interpretation of changes in rates, because preceding and subsequent trends are known. Additionally, they inform the impact of coronary risk factor reductions and improvements in acute cardiac care on the rate of admissions for MI. Globally, there are relatively few studies of population-based hospitalisation rates for MI covering more than a decade,^7–11^ and no reports of very long-term trends in England. Of those studies reporting trends of 20 years or more, age-standardised rates started to fall from the mid-1980s or 1990s,^7–11^ however, few of these studies have reported detailed age and sex stratification.

Given reports of recent upward trends in MI hospitalisation rates in younger adults, especially in women, in other countries,^6 12 13^ and also the increasing age of the English population with a likely morbidity burden associated with advancing age, detailed study of age-specific and sex-specific trends at a population level is warranted to inform coronary disease preventive efforts. The aim of this study is to analyse the timing and scale of temporal changes in rates of hospitalised MI in England by age and sex over the past 50 years.

## Methods

### Data sources

Data on hospital admissions for MI from 1968 to 2016 in England were obtained from datasets held by the Unit of Health-Care Epidemiology at the University of Oxford. National annual hospital admission data from 1968 to 1985 were collected on a one-in-ten sample basis in the Hospital In-Patient Enquiry (HIPE).^14^ It ceased temporarily between 1986 and 1989, but, from April 1989 onwards, Hospital Episode Statistics (HES) have been collected on all admissions to all hospitals in England (population ~55 million in 2016). HES data were provided by NHS Digital (https://digital.nhs.uk/data-and-information). From 1999, person-based linkage of HES became possible, which allowed successive records for the same person to be brought together in a time-sequenced record for the individual.

A regional dataset (the Oxford Record Linkage Study (ORLS)) was included, described elsewhere,^15 16^ because it is unique in England in having person-linked data from 1968. Unlike the national data, there was no sampling in the early years (ie, it is complete enumeration) and no period for which data are not available. From 1968 to 1998, the data were provided to the ORLS directly by individual hospitals and health authorities in the Oxford NHS region. From 1999, the ORLS data are the subset of English national HES that comprises records for residents of the former Oxford NHS region (population ~2.5 million in 2016). Central and South Bristol Research Ethics Committee (No 04/Q2006/176) approved the building and analysis of the linked datasets.

All admissions for MI were identified from the national and Oxford region datasets. All patients aged 15–84 years at time of admission to hospital with a primary diagnosis of MI (International Classification of Diseases (ICD) codes ICD-8 and ICD-9: 410, ICD-10: I21–I22) were included.

### Measures of hospital admissions

Three measures of annual hospital admission rates were used^16^ to capture different aspects of the changing burden from hospitalisations for MI. The first was an episode-based measure of all admissions for MI, in which every admission for every patient is counted regardless of how many admissions or transfers for each patient occurred each year (termed the ‘all-admissions rate’); this is the main measure reported, unless specified otherwise. The second measure used person-linked data to identify the number of people admitted for MI in each calendar year, counting each person once regardless of how many admissions they had in that year (termed the ‘annual-person admissions rate’). The third measure used the person-linked data to calculate the first-recorded admission for MI in the whole dataset for each individual (termed the ‘first-recorded admissions rate’). As two of the measures use person-linked data, these could be calculated from the national data only from 1999 onwards, but all three measures were available from the ORLS dataset for the entire study period (1968–2016). We also present numbers of admissions, on which the rates are based, because numbers show the absolute burden of MI.

### Statistical analyses

All analyses were conducted separately for men and women. The average age at admission is presented as mean and SD. We present age-standardised rates to summarise overall changes; underlying age-specific rates to show the important detail behind the overall rates and numbers of events to show the health-service burden of MI.

Age-standardised annual hospitalisation rates were calculated for each of the three measures for MI (all-admissions, annual-person and first-recorded). For national rates, the number of events for each measure was the numerator, and the mid-year population estimates for England were the denominator for each of the three measures. Population denominators were obtained from the Office for National Statistics for England and shown in online supplemental file 1. Regional rates for the ORLS were calculated similarly, with the population of Oxford NHS region as the denominator (online supplemental file 1). Age-standardisation of overall rates in each group was calculated by the direct method using 5-year age groups, with the 1976 European Standard Population as the standard relevant to the time period covered by this study. Rates are presented per 100 000 person-years with 95% CI. Annual age-specific rates were calculated for MI admissions for England and ORLS by 5-year age groups. The youngest age groups were combined due to small numbers (national: 15–34 years; ORLS: 15–34 for men and 15–44 years for women). Age-standardised and age-specific rates for MI admissions in England and ORLS were also calculated as average annual rates for each 3–5 year time period from 1968 to 2016.

10.1136/jech-2021-216689.supp1 Supplementary data

Because of the marked peak and subsequent decline in rates during the study period, we calculated the change in rates within two separate periods: 1968–1970 to 1981–1985, and 1990–1995 to 2012–2016. The percentage change in rates between the first and last time interval in each period was estimated from the exponential of the beta-coefficient for time interval from Poisson regression models, with the models for the youngest age group also adjusted for 5-year age group.

Trends in age-specific rates for all-admissions in both England and the Oxford region from 1968 to 2016 were analysed using log-linear joinpoint regression models to identify statistically distinct log-linear trends. A log-linear transformation provides constant percentage change in rates over time and is more robust in conditions with lower counts. Average annual percentage changes (AAPCs) for each statistically distinct period and the full period were calculated by sex and age group. The two-sided significance level was p<0.05 for all tests and adjustments were made for multiple testing. Joinpoint analyses were performed using Joinpoint Regression Programme V.4.4.0.0.

## Results

The study included 3.5 million hospital admissions for MI in England. Overall, men accounted for about two thirds (68%) of admissions (69% of HIPE, 67% of HES), and about half of the admissions in men (48%) and over two thirds of admissions in women (71%) were for those aged 65 years or older. In the regional ORLS dataset from 1968 to 2016, there were 123 891 admissions for MI, with average annual admissions of 2528. The proportion of admissions for men (69%) was similar to that seen for the national data, as was the proportion of admissions in men and women aged 65 years and over.

From 1968–1970 to 2012–2016, the mean age of men admitted with an MI in England increased from 59.7 years to 64.4 years, and for women, from 66.4 years to 69.2 years. Over the same time period, the proportion of admissions for men aged 70–84 years increased from 18% to 38% and, for women, it rose from 42% to 56%.

### Age-standardised admission rates for MI

**Figure 1:**
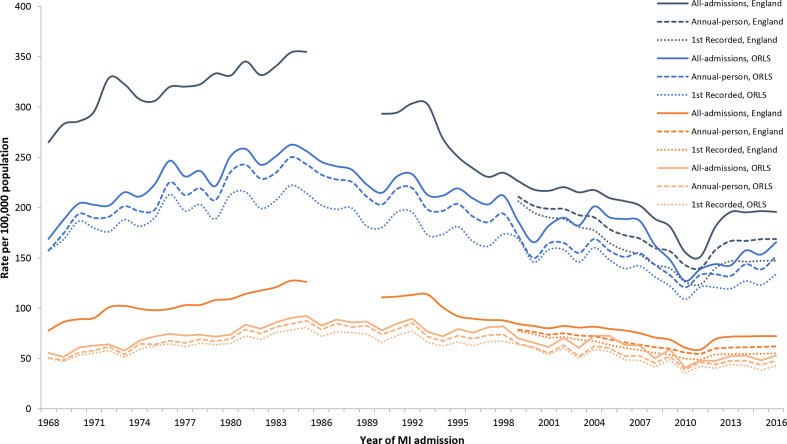
Annual age-standardised hospital admission rates for myocardial infarction per 100 000 population: England and Oxford Record Linkage Study (ORLS) area data for men (blue) and women (red), 1968–2016. MI, myocardial infarction.

Trends in age-standardised annual admission rates for MI in England and the Oxford Region are illustrated in figure 1, online supplemental file 1. From 1968 to 2016, rates fell by 31% for men and 14% for women. For men in England, the all-admission rate for MI was 265 (95% CI: 258 to 273) per 100 000 population in 1968, peaking at 355 (346 to 364) in 1985. For women, it was 78 (74 to 81) in 1968, and rose to a peak of 127 (123 to 132) in 1984. Between 1990 and 2011, rates of all-admissions for MI declined, with reductions of over a third in both men (38.8%) and in women (37.4%). From 2012 onwards, rates increased in both sexes.

From 1999 to 2016, annual-person and first-recorded admissions rates were on average 17% and 26% lower, respectively, than the all-admission rates. The trends in annual-person and first-recorded admission rates followed a similar pattern to those for all-admission rates from 1999 onwards, with reductions of 22% and 29% in annual-person and first-recorded rates, respectively, for both men and women. The Oxford Region data followed a similar pattern to the national data (figure 1); the average annual rates of all three measures of admissions in each time period for both England and the Oxford Region are shown in online supplemental file 1.

### Age-specific hospitalisation rates for MI

**Figure 2:**
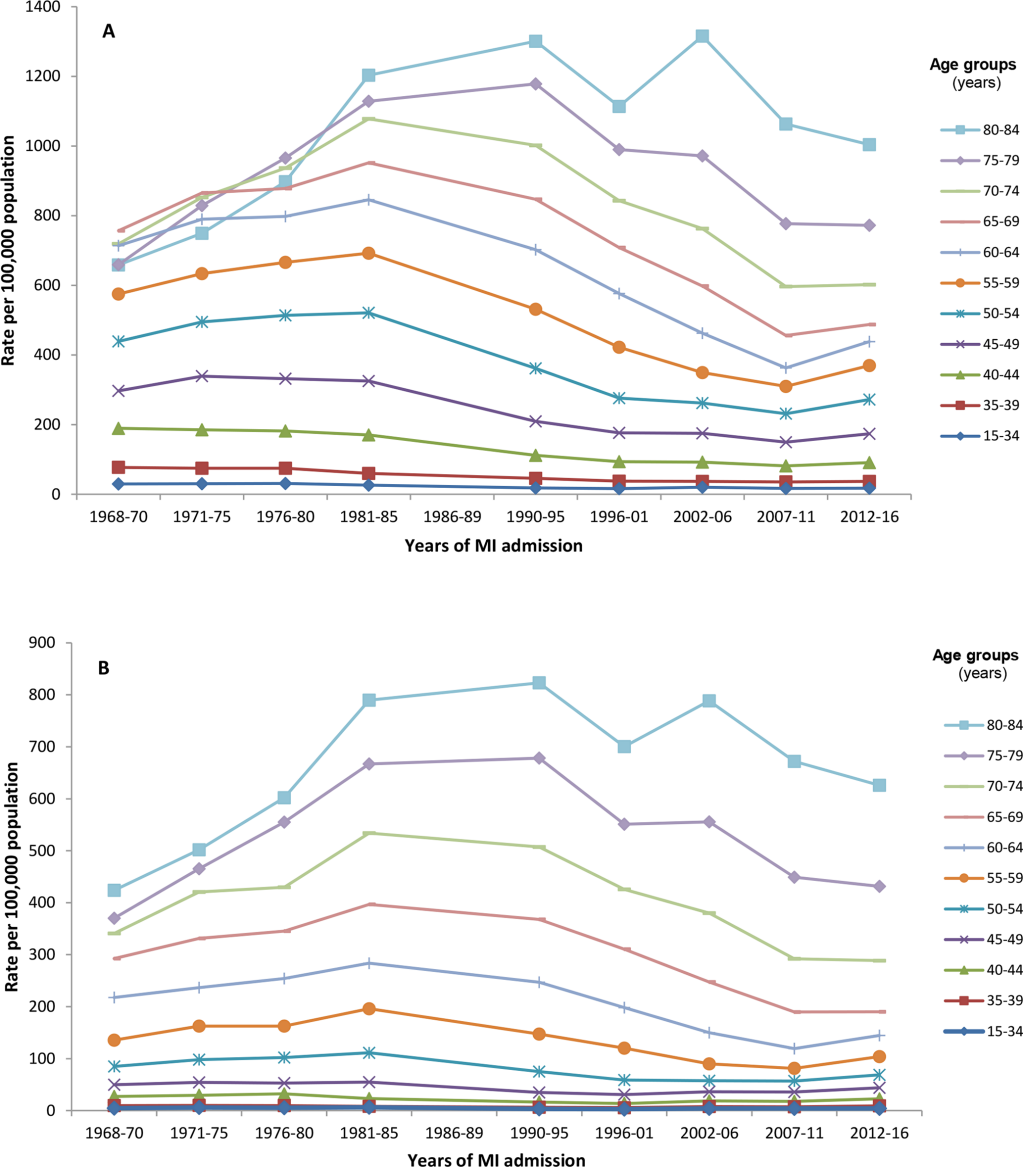
Age-specific rates of all-admissions for myocardial infarction by sex: England, 1968–2016. (A) Men. (B) Women. There were no English national data available for the period 1986–1989; 1996–2001 includes pre-linkage years (1996–1998); rates are presented in aggregated 5-year groupings for ease of visualisation. MI, myocardial infarction.

The age-specific rates of admissions for MI in men and women in England between 1968 and 2016 are shown in table 1 and represented pictorially in figure 2. Age-specific admissions rates were higher at older ages, and at any given age were consistently higher in men than women. Overall, admission rates were substantially lower at the end of the study period than at the start for all age groups, with the exception of those aged 75 years or older where rates were higher.

**Table 1:** Age-specific average annual rates of all-admissions for myocardial infarction and percentage change by sex: England, 1968–2016

	Age-specific average annual rates per 100 000 population									Change in average annual rates % (95% CI)*	
Age (years)	1968–1970	1971–1975	1976–1980	1981–1985†	1990–1995	1996–2001	2002–2006	2007–2011	2012–2016	1968–1970 to 1981–1985	1990–1995 to 2012–2016
*Men*											
15–34	29	31	31	26	18	16	18	21	18	−10.9 (−28.1 to +10.4)	−2.5 (−8.6 to +4.1)
35–39	77	74	75	60	45	38	37	35	37	−22.3 (−32.3 to −10.8)	−18.4 (−22.1 to −14.6)
40–44	189	185	182	170	112	94	92	82	91	−10.2 (−17.8 to −1.9)	−19.1 (−21.4 to −16.8)
45–49	297	339	332	325	210	177	175	150	174	9.4 (+2.4 to +17.0)	−17.2 (−18.9 to −15.5)
50–54	439	495	514	521	362	276	262	231	272	18.7 (+12.1 to +25.7)	−24.7 (−26.0 to −23.4)
55–59	575	634	666	692	532	422	350	310	370	20.4 (+14.7 to +26.4)	−30.4 (−31.5 to −29.4)
60–64	714	790	798	846	702	577	463	363	439	18.5 (+13.2 to +24.0)	−37.6 (−38.4 to −36.7)
65–69	757	865	878	952	847	708	598	456	487	25.8 (+19.7 to +32.2)	−42.5 (−43.3 to −41.7)
70–74	719	853	937	1078	1002	843	763	596	602	49.8 (+41.2 to +59.1)	−39.9 (−40.7 to −39.1)
75–79	659	829	966	1129	1178	990	972	777	772	71.2 (+58.5 to +85.0)	−34.5 (−35.4 to −33.5)
80–84	658	749	898	1203	1301	1114	1315	1063	1004	82.7 (+64.6 to +102.9)	−22.8 (−24.1 to −21.5)
*Women*											
15–34	5	6	5	6	3	3	4	4	4	32.9 (−21.3 to 124.5)	34.2 (+16.3 to +54.8)
35–39	10	10	10	8	6	6	8	7	9	−16.8 (−44.7 to +25.0)	44.9 (+30.6 to +60.9)
40–44	27	30	32	23	16	14	19	18	23	−10.7 (−30.3 to +14.2)	39.6 (+30.9 to +49.0)
45–49	50	54	53	55	35	31	36	36	44	10.0 (−7.2 to +30.3)	25.5 (+20.0 to +31.3)
50–54	85	98	102	111	75	59	57	57	69	30.4 (+14.5 to +48.5)	−8.6 (−11.8 to −5.3)
55–59	135	163	163	196	147	120	90	81	104	44.8 (+31.3 to +59.6)	−29.4 (−31.4 to −27.4)
60–64	218	236	254	284	247	198	150	119	144	30.7 (+20.7 to +41.5)	−41.6 (−42.9 to −40.2)
65–69	293	331	345	397	368	311	247	190	190	34.0 (+24.6 to +44.1)	−48.3 (−49.3 to −47.3)
70–74	341	421	430	534	507	426	380	392	289	53.7 (+43.1 to +65.1)	−43.1 (−44.1 to −42.0)
75–79	370	466	555	667	678	551	555	449	432	74.3 (+61.2 to +88.5)	−36.4 (−37.4 to −35.3)
80–84	424	502	602	790	823	700	788	672	626	84.0 (+68.1 to +101.4)	−23.9 (−25.2 to −22.7)

*Estimated from the exponential of the beta-coefficient for time period from Poisson regression models.

†There were no English national data available for the period 1986–1989.

In the earlier part of the study period (1968–1985), rates increased in all age groups except those aged less than 45 years, for which there were modest falls. Among those aged 45 years and older, the proportional increases in rates over this period were greatest in the oldest age groups. By contrast, in the later part of the study period (1990–2016), rates fell in all age groups in men, ranging from −2.5% (95% CI: −8.6% to +4.1%) in 15–34 year olds to −42.5% (−43.3% to −41.7%) in 65–69 year olds. In women, rates declined in those aged 50 years or older, ranging from −8.6 (−11.8 to −5.3) in 50–54 year olds to −48.3 (−49.3 to −47.3) in 65–69 year olds. There were statistically significant increases in rates in women aged under 50 years, although absolute rate increases were marginal.

### Joinpoint analyses

More detailed analyses using joinpoint revealed differences between ages, and between men and women, in the timing of changes in age-specific rates (table 2). For men aged 55 years or older, there was a significant rise in rates until the mid-1980s/early 1990s, after which consistent declines were seen until 2010–2011. Declines in rates started earlier in men aged <55 years, although downward trends were attenuated from the mid-1990s to around 2007. The exceptions were in the youngest age groups where rates changed direction at different time points until 2010–2011. In men aged 15–34 years, admission rates significantly increased from 1996 to 2007 followed by a decline, and in men aged 35–39 years rates increased from 2001 to 2007 and then declined. Similar patterns were observed in women except that the increasing rates in the early part of the study period were seen across a wider range of age groups including those aged less than 50 years. From 2010 to 2011 across almost all age groups, there was a significant change in the direction of the trends, with annual increases in admission rates for men aged less than 80 years starting from 2010–2011 to 2016. In women, only those in the 40–69-year age groups followed this pattern.

**Table 2:** Average annual percentage change and joinpoint trends in rates of all-admissions for myocardial infarction in England by sex, age group and overall age-adjusted: England, 1968–2016

Age (years)	AAPC 1968–2016	Period 1	APC	Period 2	APC	Period 3	APC	Period 4	APC	Period 5	APC
*Men*											
15–34	−1.0	1968–1996	−2.2*	1996–2007	2.1*	2007–2010	−15.2	2010–2016	7.2*		
35–39	−1.4†	1968–1980	−0.9	1980–2001	−3.5*	2001–2007	3.7*	2007–2010	−8.8	2010–2016	3.8*
40–44	−1.4†	1968–1981	−0.3	1981–1997	−4.2*	1997–2007	0.2	2007–2010	−7.4	2010–2016	4.6*
45–49	−1.1†	1968–1983	0.6	1983–1996	−5.0*	1996–2007	0.0	2007–2010	−7.8	2010–2016	5.5*
50–54	−0.9†	1968–1985	1.0	1985–1997	−5.4*	1997–2011	−1.2*	2011–2016	4.4*		
55–59	−0.7†	1968–1985	1.3*	1985–2000	−4.3*	2000–2011	−1.9*	2011–2016	5.9*		
60–64	−0.8†	1968–1985	1.4*	1985–2011	−3.6*	2011–2016	6.8*				
65–69	−1.0†	1968–1993	0.2	1993–1996	−6.7	1996–2007	−2.3*	2007–2010	−9.9	2010–2016	4.2*
70–74	−0.6	1968–1993	1.0*	1993–1996	−7.0	1996–2006	−1.4*	2006–2010	−7.1*	2010–2016	1.9*
75–79	0.1	1968–1993	2.0*	1993–1997	−6.6*	1997–2004	0.7	2004–2011	−4.8*	2011–2016	2.2*
80–84	0.8†	1968–1993	2.9*	1993–1997	−6.8*	1997–2004	4.0*	2004–2010	−5.3*	2010–2016	−0.2
15–84‡	−0.9	1968–1993	−0.2	1993–1996	−7.9	1996–2007	−1.2*	2007–2010	−7.8	2010–2016	4.2*
*Women*											
15–34	−0.5	1968–1998	−2.5*	1998–2003	10.9	2003–2011	−4.2	2011–2016	7.0		
35–39	−0.3	1968–1997	−2.3*	1997–2016	2.7*						
40–44	0	1968–1977	3.1	1977–1997	−4.5*	1997–2006	4.7*	2006–2010	−3.7	2010–2016	6.9*
45–49	−0.1	1968–1983	0.5	1983–1996	−4.7*	1996–2007	2.5*	2007–2011	−3.0	2011–2016	7.3*
50–54	−0.3	1968–1985	1.6*	1985–1997	−5.7*	1997–2011	0.1	2011–2016	5.3*		
55–59	−0.4	1968–1985	2.6*	1985–2001	−4.8*	2001–2010	−2.0*	2010–2016	5.9*		
60–64	−0.6	1968–1984	2.0*	1984–1992	−1.7	1992–2010	−4.4*	2010–2016	5.7*		
65–69	−0.9†	1968–1992	1.0*	1992–1995	−5.9	1995–2007	−3.1*	2007–2011	−7.1*	2011–2016	3.8*
70–74	−0.6	1968–1993	1.4*	1993–1996	−8.1*	1996–2005	−1.2*	2005–2010	−6.2*	2010–2016	1.0
75–79	0	1968–1993	2.2*	1993–1996	−9.7*	1996–2005	0.4	2005–2010	−5.6*	2010–2016	0.3
80–84	0.6	1968–1993	2.7*	1993–1996	−9.4*	1996–2005	2.2*	2005–2010	−4.7*	2010–2016	−0.7
15–84‡	−0.5	1968–1993	−0.7*	1993–1996	−8.3	1996–2007	−1.2*	2007–2010	−6.8	2010–2016	3.2*

The joinpoint analysis is used to find the best-fit line through several years of data. Line segments are joined at points called joinpoints. Each joinpoint denotes a statistically significant change in trend.

*The APC is significantly different to zero (p=0.05)

†The AAPC is significantly different to zero (p=0.05).

‡Age-adjusted.

AAPC, average annual percentage change; APC, annual percentage change.

Joinpoint analyses for person-based and first-recorded rates from 1999 to 2016 are shown in online supplemental file 1.

For men and women of all ages (15–84 years) in the Oxford region, similar trends were found for all-admission rates for MI compared with the national data (online supplemental file 1). However, age-specific trends differed, including the lower number of joinpoints in some age groups compared with the national data, the continuation of decreasing rates in the most recent years in the older age groups and no change in trend in the youngest age groups, although the rates were very low (online supplemental file 1).

### Number of admissions for MI

To demonstrate the absolute change in admissions for MI and the associated burden to health services, table 3 shows the annual average number of admissions for MI in England and percentage change by sex and age group. In 2012–2016, there were on average 70 000 admissions for MI per year (48 318 and 21 717 for men and women, respectively). The number of admissions for MI rose in the 1970s, peaked in the mid-1980s and declined thereafter until 2007–2011, after which there was a modest rise.

**Table 3:** Annual average number of admissions for myocardial infarction and percentage change by sex and age group: England, 1968–2016

	Annual average number of admissions (n)									Change in annual average numbers* (%)	
Age (years)	1968– 1970	1971– 1975	1976– 1980	1981– 1985^†^	1990– 1995	1996– 2001	2002– 2006	2007– 2011	2012– 2016	1968–1970 to 1981–1985	1990–1995 to 2012–2016
*Men*											
15–34	447	486	556	442	334	304	358	285	327	−1.1	−2.1
35–39	1117	1074	1112	1010	734	668	722	651	637	−9.6	−13.2
40–44	2843	2664	2576	2368	1836	1507	1720	1589	1663	−16.7	−9.4
45–49	4803	5066	4682	4300	3338	2761	2875	2773	3353	−10.5	0.4
50–54	6000	7526	7408	6802	4751	4348	4034	3739	4998	13.4	5.2
55–59	8440	8480	9548	9040	6496	5158	5548	4631	5855	7.1	−9.9
60–64	9437	10 528	9694	10 558	8078	6329	5744	5490	6318	11.9	−21.8
65–69	7780	9568	10 002	9528	8982	7045	6392	5242	6978	22.5	−22.3
70–74	4697	6466	8044	9288	8721	7290	6853	5663	6297	97.7	−27.8
75–79	2697	3540	4906	6380	6934	6608	6762	5754	6263	136.6	−9.7
80–84	1480	1674	2102	3318	4774	4287	6116	5306	5630	124.2	17.9
15–84	49 740	57 072	60 630	63 034	54 978	46 304	47 125	41 123	48 318	26.7	−12.1
*Women*											
15–34	70	90	96	106	60	59	78	71	80	51.4	33.3
35–39	137	140	140	136	105	108	151	139	161	−0.7	53.3
40–44	407	420	446	318	269	237	350	354	425	−21.9	58.0
45–49	830	818	744	716	556	511	601	671	865	−13.7	55.6
50–54	1223	1552	1508	1466	988	933	897	931	1285	19.9	30.1
55–59	2140	2348	2494	2670	1818	1434	1457	1243	1680	24.8	−7.6
60–64	3277	3562	3500	3940	3010	2263	1933	1871	2166	20.2	−28.0
65–69	3867	4562	4808	4810	4466	3355	2841	2325	2882	24.4	−35.5
70–74	3573	4784	5198	6276	5721	4422	3974	3103	3328	75.7	−41.8
75–79	2827	3804	5042	6290	6144	5274	5130	4121	4124	122.5	−32.9
80–84	2093	2564	3350	4854	5841	4760	5979	4918	4720	131.9	−19.2
15–84	20 443	24 644	27 326	31 582	28 976	23 356	23 392	19 747	21 717	54.5	−25.1

* Percent change of annual averages: (most recent period–earliest period)/earliest period.

† There were no English national data available for the period 1986–1989.

The overall average annual number of admissions for MI from 1968–1970 to 2012–16 reduced by 3% in men, and increased by 6% in women. When we restricted the time period to 1990–2016, the mean number of admissions per year declined by 12% in men, and 25% in women. Declines were observed in most age groups, but there were notable increases in admissions among elderly men (aged 80–84 years) and younger women (<55 years). Between 2007 and 2016 there were modest increases in both men and women at almost all ages, with the greatest increases among those in late middle age (50–69 years) in both sexes.

## Discussion

The datasets used in this study are the longest continuous run of routinely collected person-based morbidity information in England. As such, they enabled us to provide a unique record of the trajectory of trends in MI in England from 1968. There was a large rise in age-standardised admissions rates in the early years, with rates peaking in the mid-1980s, followed by a decline of over a third from 1990 onwards. In contrast, there was only a small overall decline in the annual average numbers of admissions for MI during the study period. The pattern of trends in MI admission rates was broadly similar for men and women and included downward trends from 1999 in annual-person and first-recorded rates of MI admissions as determined from record-linked data. Trends in the middle age groups underpinned these overall reductions in the more recent years. During the entire study period, the greatest variability in trends was in the oldest and youngest age groups. Men and women aged 70 years or older had the largest and most sustained rate increase in the earlier years, with a subsequent decline for most of the study period until 2010–2011. In younger women (<50 years old), there was a significant increase in rates from the mid-1990s. After 2010–2011, with the exception of the oldest age groups, rates increased in men and women with the most marked upward trends in those aged under 65 years.

### Strengths and limitations

To the best of our knowledge, this is the first study to report very long-term trends of hospitalisation rates for MI in England over five decades. The large size of our dataset allowed examination of rates by 5-year age groups in men and women separately. The analysis of unlinked all-admissions rates maximised the time period for which national rates could be assessed, and reporting annual-person and first-recorded admission rates accounted for the impact of recurrent events. A limitation of this study is that there were no national data available between 1986 and 1989. However, the Oxford Region data were available for the entire study period and followed similar patterns throughout, so it is likely that national rates also started to fall during those years. Our study spans three versions of ICD codes and each version includes specific codes for MI. However, there have been changes to coding standards, which have affected the coding of MI types, and therefore we were unable to stratify our data according to ST-elevation and non-ST-elevation MI.^17^ We cannot identify changes in hospital admission rates attributable solely to changes in patients’ healthcare-seeking behaviour and in referral practices.

### Comparison with other studies

Globally, there are few published studies of very long-term trends in hospitalisation rates for MI. The two earliest (and longest) studies were from the USA—the community-based Worcester Heart Attack Study and a study using the US National Hospital Discharge Survey. Both covered the years from the mid to late 1970s to 2005,^10 11^ and reported similar trends to ours, with rising hospitalisation rates until the early to mid-1980s. From 1981 to 2005, these studies reported reductions of 35% and 29%, respectively, which were slightly less than the declines observed in our study. Other published studies covering 20 years or more began in the late 1980s or mid-1990s and also reported an overall reduction in rates (about 2% per year).^7–9 18^

Among the studies above, three also reported age-specific estimates, although each started in a different decade.^7 9 10^ In two of these studies, only the oldest age groups demonstrated a different trend to overall rates, with increasing rates up to the mid-2000s and declining rates since,^9 10^ similar to the trends in the 75–84-year age group in our study. A Canadian study using administrative electronic hospital data for Ontario from 1994 to 2012 reported that average annual hospitalisation rates for MI declined in almost all age groups for both men and women, with the greatest decline in 65–74 year olds (41% and 47%, respectively).^7^ The exception was younger women (20–49 years) in whom rates increased by 1.2% per year, similar to other studies of incident MI over a shorter duration,^12 19^ and findings for younger women and men in our study.

### Explanations for trends

The diagnosis of MI has evolved over the 50-year study period as increasingly sensitive cardiac biomarkers have been introduced into clinical practice and the threshold for diagnosing MI has been lowered accordingly. The impact of the introduction of troponin assays in the early 2000s and the more recent high-sensitivity troponin assays has had a profound effect on increasing identification of lower severity cases of MI.^20 21^ Uniquely, we have shown an upward trend in MI hospitalisation rates from 2010 onwards. This is likely to be, in part, a result of the introduction of high-sensitivity troponin assays in English hospitals around that time, and the consequent increase in the number of cases of MI with marginally elevated troponin levels. Of the other studies that extended beyond 2010, all reported a continued decline in rates to 2014,^7 22 23^ but it is unclear when higher-sensitivity troponin assays were in use in the populations in these studies.

In contrast to the effect of diagnostic changes, reductions in coronary risk factors have contributed to the dramatic decrease in MI incidence since the mid-1980s.^24 25^ Smoking prevalence in England during this period halved,^24^ accounting for the greatest decline (about a quarter) and when combined with reductions in hypertension and hypercholesterolemia account for almost half of decline in MI incidence in a population-based cohort of English men from 1978 to 2004.^25^ However, major advances in the acute treatment of MI have led to reductions in case fatality from MI,^5^ potentially resulting in a greater pool of people at high risk for a subsequent MI and readmissions. A report from Olmsted County found a larger rate reduction for hospitalised first MIs compared with recurrent MIs,^18^ which is consistent with the greater reduction in first-recorded MIs than all MIs observed in our study.

Differences in age-specific and sex-specific trends of MI continue to be reported, and highlight the necessity of stratifying MI rates by age groups.^6 12 13^ The most consistent trends are generally found in the ‘middle age’ groups. The impact of increasing prevalence of diabetes and obesity has been postulated as a reason for adverse trends in younger age groups, however, there are limited studies supporting this hypothesis.^23 26^ Initial reports of the effect of the introduction of troponin assays indicated that additional MIs were identified in older adults.^21 27^ However, increasingly sensitive troponin assays since this diagnostic biomarker was first introduced could result in a relatively greater number of younger adults being diagnosed with MI, however, there is limited data regarding this.

### Conclusions

This study reports trends in MI hospitalisation in England over the past five decades. Despite recent substantial declines in hospital admission rates for MI, the burden of annual admissions in England remains high. Some of the potential reduction in need for healthcare provision resulting from falling age-specific rates over the later period of the study has been offset by a growing and ageing population, leading to increasing numbers of MI admissions. This has implications for health resource use and highlights the need for continued surveillance of and preventive strategies for coronary disease. Given recent changes to the diagnostic criteria for MI, future research would benefit from extending the examination of contemporary trends in MI to acute coronary syndromes. This would reduce the impact of diagnostic changes over time and facilitate comparisons with other studies. Our study also highlighted the importance of examining age-specific and sex-specific trends. The rise in MI hospitalisation rates in younger women and men is of concern, and has implications for clinicians and policy makers. Primary prevention guidelines and public awareness campaigns should continue to include the message that coronary disease is not just a disease of men and the elderly.

## Data Availability

Data may be obtained from a third party and are not publicly available. English hospital episode statistics can be obtained from NHS Digital at www.digital.nhs.uk.
