# Supplementary material for: Long-term trends in population-based hospitalisation rates for myocardial infarction in England: a national database study of 3.5 million admissions, 1968–2016
Source: J Epidemiol Community Health. 2021 Jul 12;76(1):45–52. doi: 10.1136/jech-2021-216689 (PMC8666807; doi:10.1136/jech-2021-216689)
Supplement: Supplementary data [file jech-2021-216689supp001.pdf]

Table S1. Average annual age-standardised hospital admission rates for myocardial infarction per 100,000 population shown in each time period by sex: data from national England statistics and the regional Oxford Record Linkage Study (ORLS), 1968-2016

	**England**				**Oxford record Linkage Study**			
	**Age-standardised rates/100,000**				**Age-standardised rates/100,000**			
	All-admissions	Annual-person	First-recorded	Population**, n	All-admissions	Annual-person	First-recorded	Population**, n
**Men**								
1968-70	278	-	-	17660567	187	176	171	312275
1971-75	312	-	-	17955880	213	196	185	413660
1976-80	326	-	-	18471700	237	220	203	772300
1981-85	345	-	-	18228860	255	240	212	821960
1986-89	-	-	-	-	237	224	196	863553
1990-95	286	-	-	18460317	220	206	183	980624
1996-01	235*	196	191	18825733	208	190	167	1048533
2002-06	214	186	174	19628760	191	159	150	1092700
2007-11	175	153	137	20553000	152	137	125	1145360
2012-16	193	166	146	21393420	153	140	125	1190240
**Women**								
1968-70	84	-	-	19163167	56	52	50	307245
1971-75	98	-	-	19363920	67	61	59	413020
1976-80	105	-	-	19791620	73	68	64	786420
1981-85	121	-	-	19407720	86	82	75	836180
1986-89	-	-	-	-	86	82	75	878407
1990-95	107	-	-	19575683	80	75	69	1003156
1996-01	89*	74	72	19835233	80	72	66	1070933
2002-06	80	71	66	20495440	68	58	55	1114280
2007-11	67	59	53	21273480	52	47	43	1167760
2012-16	72	61	55	21989040	51	46	42	1213640

Note: There were no English national data available for the period 1986-1989; and no person-based linked data for England prior to 1999.
* 1996-01 includes pre-linkage years (1996-98); ** average annual population per time period

Table S2. Average annual percentage change and joinpoint trends in MI hospitalisation rates for annual-person based admissions by sex, age group and overall age-adjusted: England, 1999-2016

**Age** (years)	**AAPC** 1999 - 2016	**Period 1**	**APC**	**Period 2**	**APC**	**Period 3**	**APC**	**Period 4**	**APC**
**Men**									
15–34	0.4	1999-2006	2.0	2006-2010	-9.4*	2010-2016	5.7*		
35–39	0	1999-2016	0						
40–44	-0.6	1999-2011	-1.9*	2011-2014	7.3	2014-2016	-4.0		
45–49	-0.2	1999-2007	-1.1*	2007-2010	-6.4	2010-2013	7.8	2013-2016	1.2
50–54	-0.1	1999-2010	-2.1*	2010-2016	3.7*				
55–59	-0.4	1999-2011	-2.6*	2011-2014	9.4	2014-2016	-1.0		
60–64	-1.2^	1999-2010	-4.4*	2010-2016	5.0*				
65–69	-2.2^	1999-2011	-4.5*	2011-2016	3.6*				
70–74	-2.3^	1999-2004	-2.4*	2004-2010	-5.4*	2010-2016	1		
75–79	-1.8^	1999-2003	0.4	2003-2010	-4.9*	2010-2016	0.5		
80–84	-1.2^	1999-2004	3.6*	2004-2010	-5.4*	2010-2016	-0.7		
**15-84****	**-1.1**	**1999-2004**	**-1.9**	**2004-2010**	**-4.3***	**2010-2016**	**2.9***		
**Women**									
15–34	0.5	1999-2016	0.5						
35–39	2.7^	1999-2016	2.7*						
40–44	2.2^	1999-2016	2.2*						
45–49	1.7^	1999-2011	0.4	2011-2016	5.0*				
50–54	1.2^	1999-2010	-0.4	2010-2016	4.1*				
55–59	0	1999-2008	-3.4*	2008-2016	3.9*				
60–64	-1.5^	1999-2010	-5.0*	2010-2016	5.4*				
65–69	-2.9^	1999-2011	-4.7*	2011-2016	1.8*				
70–74	-2.4^	1999-2004	-1.8	2004-2010	-5.5*	2010-2016	0.2		
75–79	-1.9^	1999-2004	0.2	2004-2010	-4.9*	2010-2016	-0.6		
80–84	-1.1^	1999-2004	2.9*	2004-2010	-4.1*	2010-2016	-1.4*		
**15-84****	**-1.2**	**1999-2004**	**-1.6***	**2004-2010**	**-4.0***	**2010-2016**	**1.9***		

AAPC: average annual percentage change; APC: annual percentage change. The joinpoint analysis is used to find the best-fit line through several years of data. Line segments are joined at points called joinpoints. Each joinpoint denotes a statistically significant change in trend.
^ The AAPC is significantly different to zero (p=0.05); * the APC is significantly different to zero (p=0.05); ** age-adjusted

Table S3. Average annual percentage change and joinpoint trends in MI hospitalisation rates for first-recorded admissions by sex, age group and overall age-adjusted: England, 1999-2016

**Age** (years)	**AAPC** 1999 - 2016	**Period 1**	**APC**	**Period 2**	**APC**	**Period 3**	**APC**	**Period 4**	**APC**
**Men**									
15–34	0.3	1999-2006	1.6	2006-2010	-9.0	2010-2016	5.3*		
35–39	-0.2	1999-2016	-0.2						
40–44	-0.8	1999-2007	-1.9*	2007-2010	-5.7	2010-2013	8.5	2013-2016	-1.7
45–49	-0.6	1999-2010	-2.8*	2010-2016	3.5*				
50–54	-0.6	1999-2010	-2.8*	2010-2016	3.6*				
55–59	-1.0	1999-2011	-3.4*	2011-2014	9.1	2014-2016	-1.2		
60–64	-1.9^	1999-2010	-5.2*	2010-2016	4.5*				
65–69	-2.9^	1999-2011	-5.3*	2011-2016	3.2*				
70–74	-3.1^	1999-2004	-3.5*	2004-2010	-6.5*	2010-2016	0.9		
75–79	-2.7^	1999-2003	-1.2	2003-2010	-5.9*	2010-2016	0.1		
80–84	-2.1^	1999-2004	1.7	2004-2010	-6.4*	2010-2016	-0.9		
**15-84****	**-2**	**1999-2010**	**-4.1***	**2010-2016**	**2.2***				
**Women**									
15–34	0.3	1999-2016	0.3						
35–39	2.4^	1999-2016	2.4*						
40–44	1.9^	1999-2016	1.9*						
45–49	1.3^	1999-2011	-0.1	2011-2016	4.7*				
50–54	0.7^	1999-2010	-1.0*	2010-2016	3.9*				
55–59	-0.5	1999-2008	-3.9*	2008-2016	3.5*				
60–64	-1.9^	1999-2010	-5.6*	2010-2016	5.1*				
65–69	-3.4^	1999-2011	-5.4*	2011-2016	1.7				
70–74	-3.0^	1999-2004	-3.0*	2004-2010	-6.1*	2010-2016	0.1		
75–79	-2.6^	1999-2003	-0.3	2003-2010	-5.2*	2010-2016	-1.0		
80–84	-1.9^	1999-2004	1.1	2004-2010	-4.9*	2010-2016	-1.3		
**15-84****	**-2.0**	**1999-2010**	**-3.8***	**2010-2016**	**1.3**				

AAPC: average annual percentage change; APC: annual percentage change. The joinpoint analysis is used to find the best-fit line through several years of data. Line segments are joined at points called joinpoints. Each joinpoint denotes a statistically significant change in trend.
^ The AAPC is significantly different to zero (p=0.05); *the APC is significantly different to zero (p=0.05); ** age-adjusted.

Table S4. Age-specific rates of all-admissions for myocardial infarction and percentage change by sex and age group: Oxford Region, 1968-2016

Age groups (years)	Age-specific average annual rates per 100,000 population[a]										Change in average annual rates, % (95% CI)[a]	
	**1968-1970**	**1971-1975**	**1976-1980**	**1981-1985**	**1986-1989**	**1990-1995**	**1996-2001**	**2002-2006**	**2007-2011**	**2012-2016**	1968-70 to 1981-85	1990-95 to 2012-16
**Men**												
15-34	13	16	321	19	16	15	14	20	13	12	+51.9 (-19.4, +186.4)	-13.9 (-37.7, +19.0)
35-39	45	37	47	45	36	35	33	32	30	24	+0.2 (-29.9, +43.3)	-31.8 (-45.6, -14.6)
40-44	81	136	124	113	95	72	67	85	75	68	+39.3 (+6.5, +82.3)	-5.2 (-17.7, +9.1)
45-49	192	213	238	234	177	144	135	149	125	134	+22.1 (+1.8, +46.4)	-7.3 (-16.1, +2.5)
50-54	263	346	349	355	324	257	212	229	191	206	+35.2 (+15.2, +58.7)	-19.6 (-26.1, -12.5)
55-59	442	448	479	486	451	401	292	312	279	297	+9.8 (-3.5, +24.9)	-26.0 (-31.3, -20.3)
60-64	492	529	593	632	599	527	425	432	329	347	+28.3 (+13.0, +45.8)	-34.3 (-38.8, -29.5)
65-69	496	580	678	741	641	692	504	561	414	402	+49.3 (+29.5, +72.2)	-41.9 (-45.7, -37.9)
70-74	514	612	717	810	817	808	616	676	509	495	+57.6 (+32.9, +87.0)	-38.8 (-42.9, -34.4)
75-79	560	575	659	897	954	993	762	829	632	623	+60.1 (+30.2, +96.9)	-37.2 (-41.7, -32.4)
80-84	505	568	797	972	1065	1086	917	1097	908	797	+92.3 (+44.6, +155.8)	-26.6 (-32.4, -20.2)
**Women**												
15-44	23	24	24	17	15	19	21	27	22	24	-29.2 (-58.8, +21.7)	+26.0 (-2.5, +62.7)
45-49	25	34	37	30	28	24	24	33	25	29	+22.9 (-25.9, +104.0)	+18.6 (-5.7, +49.3)
50-54	46	67	55	68	64	44	42	48	44	50	+47.3 (+0.8, +115.3)	+15.0 (-4.8, +38.9)
55-59	63	108	100	122	126	102	67	73	64	64	+93.3 (+39.8, +167.4)	-37.3 (-46.3, -26.9)
60-64	141	146	149	187	201	176	141	125	85	100	+32.3 (+5.4, +66.2)	-43.1 (-49.8, -35.4)
65-69	195	216	256	279	290	284	223	226	146	131	+43.5 (+16.5, +76.7)	-53.9 (+58.6, -48.6)
70-74	270	316	354	412	391	393	317	330	242	208	+52.7 (+25.7, +85.5)	-46.9 (-51.9, -41.5)
75-79	318	336	416	558	565	548	437	445	370	311	+75.5 (+43.0, +115.5)	-43.4 (-48.3, -38.0)
80-84	345	391	574	714	643	652	544	635	509	530	+107.2 (+62.8, +163.8)	-18.8 (-25.4, -11.7)

[a] 'Estimated from the exponential of the beta-coefficient for time period from Poisson regression models.

CI, confidence interval.

Figure S1. Age-specific rates of all-admissions for myocardial infarction by sex: Oxford Region, 1968-2016

a) Men

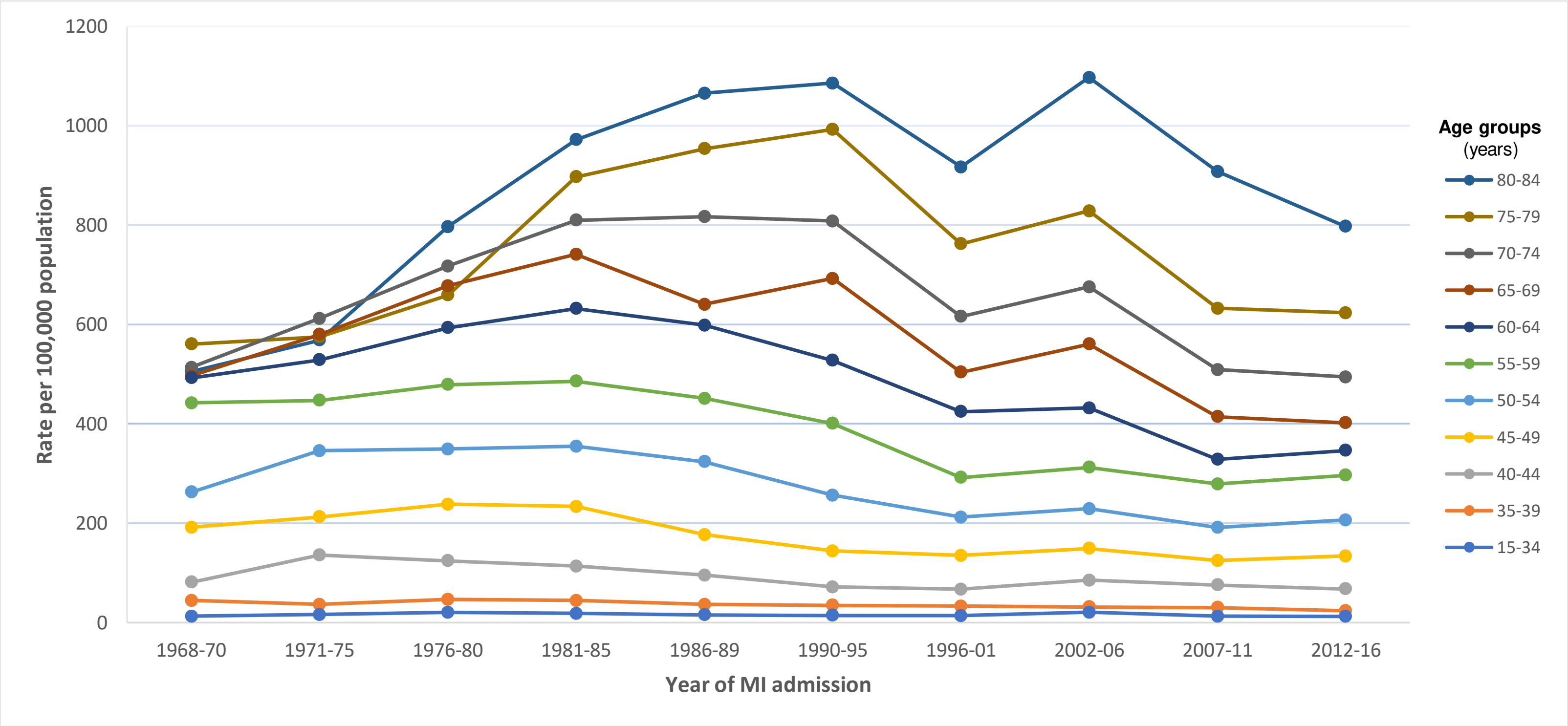

Wright FL, *et al. J Epidemiol Community Health* 2021; 76:45–52. doi: 10.1136/jech-2021-216689

b) Women

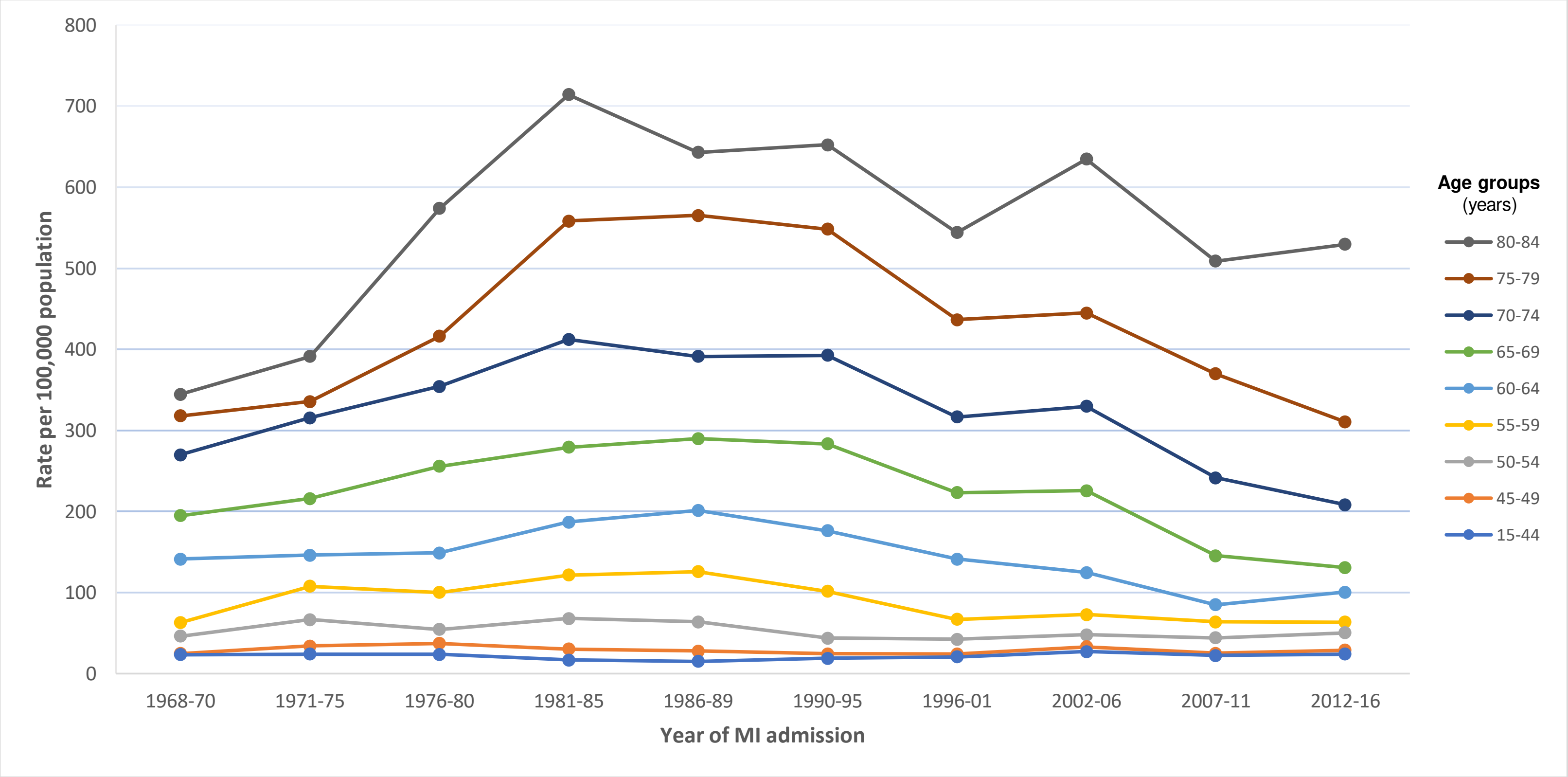

Wright FL, *et al. J Epidemiol Community Health* 2021; 76:45–52. doi: 10.1136/jech-2021-216689

Table S5. Average annual percentage change and joinpoint trends in admission rates for myocardial infarction by sex, age group and overall age-adjusted: Oxford Region 1968-2016

**Age** (years)	**AAPC** 1968 - 2016	**Period 1**	**APC**	**Period 2**	**APC**	**Period 3**	**APC**	**Period 4**	**APC**	**Period 5**	**APC**
**Men**											
15–34	-0.2	1968-2016	-0.2								
35–39	-1.3^	1968-2016	-1.3*								
40–44	-0.7	1968-1978	3.2	1978-1994	-4.3*	1994-2007	2.3*	2007-2010	-12.5	2010-2016	3.0
45–49	-0.6	1968-1982	1.7*	1982-1993	-5.3*	1993-2007	0.7	2007-2010	-10.1	2010-2016	4.6*
50–54	-0.6	1968-1983	1.6*	1983-1996	-3.8*	1996-2007	0.2	2007-2010	-11.3	2010-2016	5.8*
55–59	-0.6	1968-1984	0.7	1984-2011	-2.3*	2011-2016	4.3				
60–64	-0.6	1968-1984	1.6*	1984-2007	-2.0*	2007-2010	-10.5	2010-2016	4.3*		
65–69	-0.1	1968-1981	3.4*	1981-2007	-1.3*	2007-2011	-11.7	2011-2016	7.4*		
70–74	0	1968-1985	2.9*	1985-2006	-1.3*	2006-2010	-9.4*	2010-2016	3		
75–79	0.3	1968-1990	3.4*	1990-2016	-2.2*						
80–84	0.9^	1968-1984	5.2*	1984-2005	0.1	2005-2016	-3.3*				
**15-84****	**-0.3**	**1968-1984**	**1.9***	**1984-2000**	**-2.0***	**2000-2007**	**0.1**	**2007-2010**	**-11.8***	**2010-2016**	**4.0***
**Women**											
15–44	1.1^	1968-2016	1.1*								
45–49	0	1968-1997	-1.8*	1997-2004	6	2004-2012	-6.4	2012-2016	18.2*		
50–54	0	1968-1986	1.8	1986-1994	-6.4	1994-2016	0.9				
55–59	-0.9	1968-1985	2.2	1985-2016	-2.6*						
60–64	-0.9^	1968-1989	2.4*	1989-2016	-3.4*						
65–69	-0.8	1968-1987	2.3*	1987-2006	-1.7*	2006-2010	-13.7	2010-2016	1.8		
70–74	-1.0^	1968-1995	1.1*	1995-2016	-3.7*						
75–79	0.2	1968-1987	4.3*	1987-2016	-2.4*						
80–84	0.9^	1968-1984	5.1*	1984-2016	-1.1*						
**15-84****	**-0.1**	**1968-1985**	**2.8***	**1985-2006**	**-1.5***	**2006-2010**	**-8.1**	**2010-2016**	**2.0**		

AAPC: average annual percentage change; APC: annual percentage change
^ The AAPC is significantly different to zero (p=0.05); * The APC is significantly different to zero (p=0.05); ** age-adjusted
The joinpoint analysis is used to find the best-fit line through several years of data. Line segments are joined at points called joinpoints. Each joinpoint denotes a statistically significant change in trend.
